# Development of postural stability in children with autism spectrum disorder: a cross-sectional study

**DOI:** 10.1080/23335432.2021.1968316

**Published:** 2021-08-20

**Authors:** Yumeng Li, Ting Liu, Carrie E. Venuti

**Affiliations:** Department of Health and Human Performance, Texas State University, San Marcos, TX, USA

**Keywords:** Postural stability, balance, nonlinear analysis, neurodevelopmental disorders, growth

## Abstract

The purpose was to investigate the effects of age on postural stability for children with autism spectrum disorder (ASD). Twenty-nine children with mild ASD were assigned into one of the three groups: 6–8 years (U8), 9–11 years (U11) and 12–14 years (U14). Participants stood barefoot with both feet on a force platform and maintained stationary for 15 seconds during eyes-open and eyes-closed conditions. Center of pressure data were collected and variables were calculated, including displacements, total distances, sway areas, and sample entropy. The variables were compared among the three groups using a mixed-model ANOVA. The *age group* effect was significant for mediolateral center of pressure displacement (*p = *0.04) and sway distance (*p = *0.02). Post-hoc comparisons revealed that U8 exhibited greater mediolateral displacement and total distance compared to U14, regardless of test conditions. The U14 group exhibited improved mediolateral postural stability compared to U8, whereas no differences were found between U8 and U11 or between U11 and U14. This may suggest that children with ASD could slowly develop postural stability but only demonstrate significant changes over a long period of time. Early intervention programs aimed to improve complexity of postural control could be beneficial.

## Introduction

Autism spectrum disorder (ASD) is a group of neurological developmental disorders that typically last throughout an individual’s lifetime. In early research, the prevalence rate of ASD in the general population was reported to be 0.2% (Williams et al. 2006) and has been growing steadily for years (Matson and Kozlowski 2011). The recent ASD prevalence rate has been estimated to be 2.5% (Elsabbagh et al. 2012; Randall et al. 2015; Zablotsky et al. 2015). Children with ASD often exhibit persistent deficits in social communication and social interaction, restricted interest or activities, and repetitive patterns of behavior (American Psychiatric Association 2013). They also display unusual responses to sensory input (e.g. visual, auditory, taste or smell sensitivity) (Wiggins et al. 2009). In addition, motor behavior symptoms have been observed in children with ASD, including motor coordination abnormalities (Fournier et al. 2010a) and poor motor functional performance (Kaur et al. 2018).

Appropriate motor function and the ability to maintain an upright body posture are crucial for balance and participation in physical activities: children with better postural stability are more likely to participate in physical activities and less likely to live a sedentary lifestyle (Lim et al. 2017). Postural control can be challenging for young children because it depends on maturation of the relevant structures (e.g. sensory system) as well as motor experiences (Cdsc et al. 2018). Previous research has generally agreed that the development of the structures responsible for postural control is completed by the age of seven (Shumway-Cook and Woollacott 1985; Assaiante et al. 2005). Children are still gaining motor experiences and developing postural control strategies after the age of seven (Cdsc et al. 2018). When testing in challenging situations, such as manipulations of visual or vestibular input, children may not attain adult levels of postural stability until the age of 15 (Cumberworth et al. 2007).

Impaired postural stability in children with ASD is well established in the literature (Lim et al. 2017). Because force plates are the gold standard for measuring biomechanical factors in the kinetic control of balance, many previous studies have utilized force plates to examine center of pressure (COP). Children with ASD have demonstrated greater COP sway displacements (Smoot Reinert et al. 2015), sway areas (Doumas et al. 2016), standard deviations of COP coordinates (Doumas et al. 2016), sway velocities (Morris et al. 2015), and root mean square of COP coordinates (Memari et al. 2013) compared to age-matched typical developing children. The majority of studies have observed greater COP sway magnitudes in children with ASD, while other studies found no differences during the eyes-open condition (Doumas et al. 2016) or all testing conditions (Greffou et al. 2012). To complement traditional linear analyses, recently, there has been increased interest in utilizing nonlinear analyses. Nonlinear analyses have been used to assess postural stability in clinical settings because diseases and disorders can be viewed as a breakdown of nonlinear feedback loops along with altered physiological complexity (Lipsitz and Goldberger 1992). An optimal sway complexity has been considered as a healthy postural control mechanism. Two studies investigated postural control complexity in ASD using multiscale entropy analysis; however, the findings were not in agreement (Fournier et al. 2014; Li et al. 2019) possibly because of different ages and severity in ASD of participants.

Previous research has demonstrated that ASD could result in delayed development or even permanent impairments of postural stability (Lim et al. 2017). Though age has been associated with gross motor skills in children with ASD (Mache and Todd 2016), development of postural control in ASD is still not well understood. To our knowledge, studies examining age-related changes in postural stability of children with ASD are still very limited. One early study tested 79 high-functioning (without intellectual disabilities) individuals with ASD between the ages of 5 and 52 during sensory organization tests (Minshew et al. 2004). Researchers observed that postural stability in individuals with ASD did not show improvement until age 12 and never achieved the level of healthy adults (Minshew et al. 2004).

The most recent guideline (i.e. Diagnostic and Statistical Manual of Mental Disorder fifth edition: DSM-5) combines all previous sub-diagnoses of DSM-IV (e.g. autistic disorder, Asperger syndrome, pervasive developmental disorder not otherwise specified (PDD-NOS) etc.) (Christiansz et al. 2016). Children with PDD-NOS based on DSM-IV are less likely to receive a DSM-5 diagnosis of ASD (Christiansz et al. 2016). Due to possible inconsistencies in ASD diagnosis and sample characteristics, more studies with ASD patients diagnosed based on the new DSM-5 are needed to confirm previous findings.

Nonlinear dynamic analyses (e.g. postural sway complexity) are also necessary because the development of postural control is a dynamic process through which children learn to control multiple degrees of freedom of body segments to maintain balance (Harbourne and Stergiou 2003). In recent years, many studies have investigated COP complexity during quiet standing tasks in different populations, because COP display highly irregular and nonstationary fluctuations (Borg and Laxåback 2010; Rigoldi et al. 2013; Fournier et al. 2014; Busa et al. 2016). Moreover, postural sway complexity can reflect the adaptive capacity of the postural control system (Li et al. 2019), thus potentially better revealing sensorimotor impairments in autistic children when processing altered visual inputs (e.g. eyes-closed condition). A thorough understanding of the age effect on ASD postural stability might facilitate better insight into the mechanisms and deficits in ASD postural control development. Furthermore, it may help in developing training or rehabilitation programs to improve postural stability in children with ASD at different ages.

Therefore, the purpose of the present study was to investigate the age effect on postural stability in children with ASD. Specifically, we compared the amplitude and complexity of COP sway during quiet standing in children with ASD among three different age groups: 6–8 years (under 8: U8), 9–11 years (U11), and 12–14 years (U14). Because of motor development, we hypothesized that children in older groups would exhibit better postural stability demonstrated by lower COP sway amplitudes and higher COP sway complexity.

## Methods

### Participants

Based on our preliminary study measuring COP data and a priori power analysis (α = 0.05, power = 0.80, Cohen’s d = 0.30, number of age groups = 3), 24 participants were needed for the total sample size. In the present study, 29 participants (aged 6 to 14 years) with level one ASD (mild) were recruited from the local autism summer camp. Based on DSM-5, participants with level one ASD could exhibit deficits in social communication without supports in place, inflexibility of behavior, and difficulty switching between activities (American Psychiatric Association 2013). Participants were included in this study if they met the following criteria: (1) had been diagnosed with level one ASD by a physician or a licensed psychologist using DSM-5 (American Psychiatric Association 2013), (2) had the ability to understand and communicate with the investigators, and (3) had the ability to perform standing tasks. The exclusion criteria included chronic medical disorders, visual impairments, current medications, and physical impairments that could affect postural stability. Participants were also excluded if they had prior or current training that specifically aimed to improve balance. Participants were assigned to one of the three groups based on their ages: 6–8 years (U8), 9–11 years (U11) and 12–14 years (U14). The two-year age window was selected to allow for a similar sample size in each group in the present study and reveal age-related developmental changes in postural control. Participant characteristics are presented in Table 1. A health control group was not recruited based on two reasons. First, reduced postural stability in ASD compared to health controls is well established in the literature. Second, the primary purpose of the study was to investigate age-related postural control developments in ASD individuals only.

**Table 1.** Participant characteristics: mean (standard deviation)

Variable	U8	U11	U14
Sample Size	9 (F = 2, M = 7)	12 (F = 5, M = 7)	8 (F = 1, M = 7)
Body mass (kg)	29.1 (12.7)	44.2 (14.1)	71.3 (18.3)
Height (m)	1.23 (0.10)	1.46 (0.07)	1.74 (0.11)
Age (yr)	6.8 (1.0)	10.4 (0.9)	13.1 (0.6)

### Data collection

Informed consent was obtained from each participant’s parents or legal guardians before data collection and verbal assent was provided by each participant. The study protocol was approved by the Institutional Review Board (IRB approval #: 6415).

The testing took place at the summer camp. Participants’ anthropometric data were measured including height and body mass. Participants were instructed to wear comfortable clothes, stand barefoot with both feet on a portable force platform, and maintain stationary for 15 seconds during eyes-open and eyes-closed conditions. The eyes-closed condition was used because it could reflect postural control impairments when processing altered visual inputs. It was difficult for young children with ASD to remain focused and maintain still for a long period. Therefore, like many previous studies in children (Ferdjallah et al. 2002; Stins et al. 2009; Fournier et al. 2014; Victorio and Fujisawa 2019), a relatively short test duration (15 seconds) was utilized. A recent study also suggested that 15-second sampling duration maintained acceptable construct and concurrent validity (Tracy et al. 2019). Participants were instructed to keep their arms relaxed at their sides. Exact foot placement was not controlled to enable participants to adopt a natural stance to more closely reflect their postural control strategies in a natural environment. COP data were collected with the force platform at 100 Hz (HUMAC® Balance System, Computer Sports Medicine, Inc., Stoughton, MA, USA). Two acceptable trials were carried out for each test condition. For each acceptable trial, the criteria included: (a) keeping eyes open or eyes closed for 15 seconds; (b) no obvious voluntary movement (e.g. looking around, swinging arms, leaning towards one side or another); (c) participants remaining quiet.

### Data analysis

The validity and reliability of the portable force plate for measuring the COP were examined based on previously published protocol (Walsh et al. 2006). A sub-sample of children with ASD (n = 5) was used to determine the validity of the portable force plate. Several COP variables (displacement, total distance and sway area) were calculated and compared to those measured by a standard floor imbedded laboratory force plate (OR6-6 model, Advanced Mechanical Technology, Inc., Watertown, MA, USA). Paired t-tests (Table 2) indicated that the differences in COP variables between the two force plates were minimal (difference = 1.4–7.3%; *t = *0.04–1.95; *p = *0.12–0.90). Test–retest intra-class correlations were calculated to assess the reliability. The intra-class correlation coefficients were satisfactory (α = 0.89–0.96). Because of the high validity and reliability of our portable force plate, no device-specific calibrations were performed as suggested in previous research (Koltermann et al. 2017).

**Table 2.** Portable force plate validity and reliability

	Portable force plate: mean (SD)	Floor imbedded force plate: mean (SD)	Mean difference	Paired t tests (p-value)	Intra-class correlation coefficient (p-value)
ML Disp (cm)	7.1 (3.7)	7.0 (3.0)	1.4%	0.04 (0.90)	0.93 (0.00)
AP Disp (cm)	6.9 (2.2)	6.4 (1.9)	7.2%	1.95 (0.12)	0.96 (0.00)
Total distance (cm)	50.5 (22.4)	52.2 (18.4)	1.9%	0.37 (0.73)	0.89 (0.01)
Sway area (cm^2^)	13.9 (10.8)	15.0 (11.1)	7.3%	0.52 (0.62)	0.93 (0.01)

Note: ML = mediolateral; AP = anteroposterior; Disp = displacement;

The COP data were filtered using a fourth-order low-pass Butterworth filter at 10 Hz. The cutoff frequency was determined using residual analyses. Linear displacements of COP (maximum values minus minimum values) were calculated in the mediolateral and anteroposterior directions. The total distance was calculated as the total length of COP sway path. The COP sway area was computed using a 95% confidence interval elliptical area. The sample entropy was calculated to assess the complexity of the COP in each direction. The sequence length (m) was 2, and the matching tolerance (r) was 0.15 times the standard deviation of COP data. These COP variables were selected because they could quantify the magnitude and complexity of postural sway and have shown differences between ASD and control groups according to previous research. (Molloy et al. 2003; Graham et al. 2015; Smoot Reinert et al. 2015; Li et al. 2019) All COP variables were calculated during each trial, and average values between the two trials were generated for statistical purposes. Customized MATLAB programs (MathWorks, Inc., Natick, MA, USA) were developed for calculating all COP variables.

No outliers were detected using Chauvenet’s criterion. The normality of COP variable distribution was assessed using Shapiro–Wilk tests. All COP variables exhibited normal distributions (Shapiro–Wilk Statistics = 0.94–0.98, *p = *0.12–0.90). The COP variables were compared among the three age groups using mixed-model ANOVA. The within-subject effect was the test *condition* (eyes-open and eyes-closed) and the between subject effect was the *age group* (U8, U11, and U14). Tukey’s honest significant difference (HSD) post-hoc tests were performed if the results of ANOVA were significant. The significance level was selected as p < 0.05. Partial ω^2^ was calculated as the measure of effect size. Statistical analyses were conducted using SPSS^TM^ (IBM Corporation, Armonk, NY, USA).

## Results

**Figure 1.**
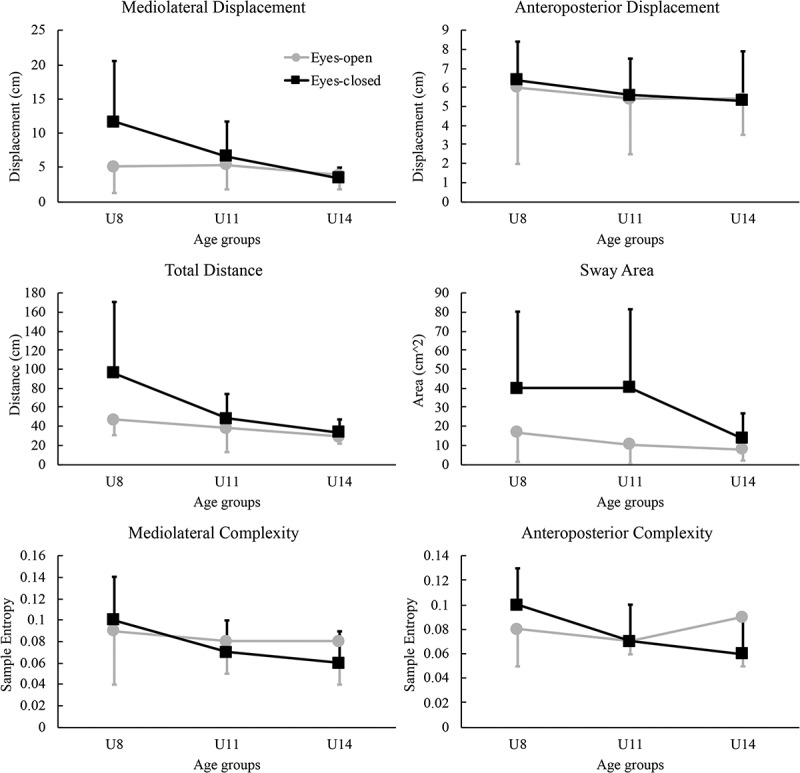
Means and standard deviations of center of pressure variables during eyes-open and eyes-closed conditions

The COP outcomes of the three age groups during eyes-open and eyes-closed conditions are shown in Table 3 and Figure 1. Based on statistical analyses, the *age group* effect was significant for mediolateral COP displacement (*F_2,26_ = *3.68, *p = *0.04, Partial ω^2^ = 0.15) and total COP sway distance (*F_2,26_ = *4.70, *p = *0.02, Partial ω^2^ = 0.20). Post-hoc comparisons revealed that the group U8 exhibited greater mediolateral displacement (95% confidence interval difference: 0.3–9.1 cm, *p* = 0.03) compared to U14, regardless of test conditions. In addition, U8 showed greater COP total distance (95% confidence interval difference: 6.1–72.5 cm, *p* = 0.02) compared to U14. However, no significant differences were observed between U8 and U11 or between U11 and U14. Other COP variables did not display significant *age group* effect (*p* = 0.13–0.71). The interaction effect of *age group* by *condition* was nonsignificant for all COP variables (*p* = 0.09–0.42).

**Table 3.** Means (standard deviations) of center of pressure (COP) variables during eyes-open (EO) and eyes-closed (EC) conditions

Variables	Conditions	U8	U11	U14	Age group Effect	Condition effect	Age group by condition
ML Disp (cm)	EO	5.1 (3.8)	5.4 (3.7)	3.9 (2.2)	*P* = 0.04*	*P* = 0.05*	*P* = 0.09
	EC	11.6 (8.9)	6.6 (5.0)	3.4 (1.6)			
AP Disp (cm)	EO	6.0 (4.0)	5.4 (2.9)	5.4 (1.9)	*P* = 0.71	*P* = 0.76	*P* = 0.94
	EC	6.4 (2.0)	5.6 (1.9)	5.3 (2.6)			
Total distance (cm)	EO	45.6 (15.4)	38.1 (24.7)	29.1 (7.8)	*P* = 0.02*	*P* = 0.01*	*P* = 0.09
	EC	95.8 (74.6)	48.4 (25.4)	33.4 (14.1)			
Sway area (cm^2^)	EO	16.9 (15.6)	10.5 (10.4)	7.9 (5.9)	*P* = 0.36	*P* = 0.00*	*P* = 0.28
	EC	39.9 (40.4)	41.3 (41.4)	13.7 (13.2)			
ML SampEn	EO	0.09 (0.05)	0.08 (0.03)	0.08 (0.04)	*P* = 0.26	*P* = 0.45	*P* = 0.42
	EC	0.10 (0.04)	0.07 (0.03)	0.06 (0.03)			
AP SampEn	EO	0.08 (0.03)	0.07 (0.01)	0.09 (0.04)	*P* = 0.13	*P* = 0.87	*P* = 0.14
	EC	0.10 (0.03)	0.07 (0.03)	0.06 (0.03)			

Note: ML = mediolateral; AP = anteroposterior; Disp = displacement; SampEn = sample entropy

The *condition* effect was significant for several variables including mediolateral displacement (*F_1,26_ = *4.01, *p = *0.05, Partial ω^2^ = 0.13), total distance (*F_1,26_ = *7.55, *p = *0.01, Partial ω^2^ = 0.21) and sway area (*F_1,26_ = *10.04, *p = *0.00, Partial ω^2^ = 0.24). All groups had greater values of mediolateral displacement, total distance and sway area during the eyes-closed conditions compared to eyes-open conditions. Other COP variables exhibited nonsignificant *condition* effect (*p* = 0.45–0.87).

## Discussion

Our study examined the age effect on postural stability in children with ASD by assessing the amplitude and complexity of COP sway during quiet standing among three different age groups. We have hypothesized that the older groups would exhibit greater postural stability compared. However, our hypotheses were only partially supported by the findings. Some age-related changes in postural control were observed between the U8 and U14 groups, while no significant differences were found between U8 and U11 or between U11 and U14.

Age-related postural changes were only seen between the U8 and U14 groups. These changes were demonstrated by reduced COP mediolateral sway displacement and total distance in the U14 group, which may indicate improved mediolateral postural stability (Li et al. 2019). The developments (i.e. reduced mediolateral sway) could indicate a more mature postural control pattern, because greater mediolateral sway has been associated with immature control of posture (Fournier et al. 2010b). Interestingly, the anteroposterior COP sway displacement did not demonstrate any age-related changes. This is inconsistent with the findings for typically developing children who demonstrated reduced anteroposterior COP displacements in older age groups (Cdsc et al. 2018). The discrepancy between the sway directions could be attributed to different postural control mechanisms. During a side-by-side stance, mediolateral sway is primarily controlled by limb loading and unloading completed through the hip mechanism (i.e. hip abduction and adduction moments) (Winter 1995; Winter et al. 1996). Anteroposterior sway is regulated by the ankle mechanism (ankle dorsiflexion and plantarflexion moments) (Winter 1995). Our findings may suggest that children with ASD could develop postural control strategies through the hip mechanism from age 8 to 14, but the ankle strategy may remain unchanged. The development of mediolateral postural stability could potentially reduce the risk of fall and facilitate participation in physical activities because impairments in mediolateral stability have been particularly associated with the risk of falling (Maki et al. 1994; Brauer et al. 2000).

In contrast to the mediolateral sway displacement and total distance, no age-related difference was observed for sway area. The sway area is a commonly utilized measure to assess the magnitude of COP sway and has been reported to be greater in children with ASD compared to age-matched healthy controls (Radonovich et al. 2013; Mache and Todd 2016). In the present study, the nonsignificant difference in sway area among age groups could be attributed to large variations within each group. Qualitatively, in older-age groups we observed a pattern of decreased sway area with a medium effect size (partial ω^2^ = 0.06). However, the large within-group variations (Table 3: standard deviations were close to mean values of sway area) could have decreased the *F* value (ratio of between-group variability to within-group variability) and resulted in nonsignificant between-group differences.

Age-related postural control changes were not observed between U8 and U11 or between U11 and U14. However, qualitatively, most COP magnitude variables displayed a trend of decreases in older-age groups with medium to large effect sizes (partial ω^2^ = 0.07–0.20). This may suggest that children with ASD could progressively develop postural stability but only demonstrate significant changes when a long period of time is analyzed (e.g. from age 6 to 14). Compared to typically developing children who had shown significantly improved postural stability every two years (Cdsc et al. 2018), children with ASD may exhibit a slower rate of development. Previous research demonstrated that children with ASD did not start to improve postural stability until age 12 and achieved a similar improvement rate as healthy controls from age 12 to 20 (Minshew et al. 2004). However, our findings were not in agreement with the previous study. Several factors could contribute to this discrepancy. First, different samples of participants were tested (i.e. high functioning in the previous study vs. mild ASD in the present study). Second, the previous study performed sensory organization tests using the NeuroCom® system (NeuroCom International Inc., Clackamas, OR), whereas we tested quiet standing using a force plate. Lastly, different statistical analyses were employed to assess the effect of age (i.e. regression vs. ANOVA). Because of the large age range (5–52 years) used in the previous study, a nonlinear curve (regression) could have been over-smoothed to fit all data but potentially distorted the pattern in the young age range (e.g. 6–14 years).

Complexity of COP sway was used in the present study to characterize the nonlinear dynamics of physiological processes during postural control. In general, lower complexity could indicate a more repetitive and restricted sway pattern that may be more vulnerable to external perturbations (Borg and Laxåback 2010), whereas greater complexity represents a more dynamic, resilient and adaptive postural control system (Wayne et al. 2014). A lack of complexity in postural control has been suggested to be early marker of developmental disabilities (Dusing and Harbourne 2010). Though some conflicting findings have been reported, complexity in postural sway has been shown to be partially compromised in children with ASD compared to healthy controls (Fournier et al. 2014; Li et al. 2019). The reduced COP complexity could be attributed to decreased complexity in the brain activity affecting the integration of sensory information and execution of movement (Goldman et al. 2009; Liu et al. 2017). In the present study, no significant differences in COP complexity were found among age groups. The nonsignificant group difference indicates that postural control adaptability remains unchanged from age 6 to 14 possibly due to brain dysfunction (e.g. dysfunction in basal ganglia and motor cortex) (Kohen-Raz et al. 1992). Because adaptability or variability in postural control serves as a foundation for development of functional skills, enhancing complexity could lead to functional changes and improvement in motor function (Dusing and Harbourne 2010). Therefore, we suggest that early therapeutic interventions that focus on improving complexity of postural control could facilitate development of postural stability and motor function for children with ASD. Interventions could include providing opportunities to experience different movements or body positions requiring various postural control strategies. Future research is needed to investigate deficits in postural control complexity for young autistic children and evaluate the efficacy of early interventions to enhance postural stability.

There are some potential limitations in the present study to consider when interpreting the results. First, the present study is using a cross-sectional design. Because different individuals were tested in each age group, some factors other than age may not be strictly controlled. For example, the intelligence quotient of participants was not strictly controlled. According to previous research, the intelligence quotient scores had a small but significant effect on postural stability (Minshew et al. 2004). However, all participants recruited in the present study had level one autism (mild) and were able to complete a series of motor competence assessments (i.e. Bruininks–Oseretsky Test of Motor Proficiency-2 (Bruininks et al. 2007) and Movement Assessment Battery-2 (Henderson et al. 2007)). Second, participation in physical activity was not listed in the inclusionary or exclusionary criteria. Different levels of physical activity participation could lead to different postural stability. However, we tried to minimize the influence of physical activity level by excluding participants with physical impairments. Moreover, all participants were able to complete a series of physical activities (e.g. running, chasing, jumping, landing, throwing and catching a ball) in the autism summer camp. Third, the sample size of females and males is different among groups. However, through qualitative comparisons, no obvious differences in postural stability variables were found between sex. Fourth, a 15-second sample duration may be a limitation for sample entropy analysis, because sample entropy of a longer duration (i.e. 1 minute) displayed a better ability to discriminate between groups (Montesinos et al. 2018). However, it was very difficult for young children with ASD to remain focused and maintain still for a long period. Lastly, during eyes-open test conditions, participants’ vision was not controlled, which could potentially influence postural sway patterns. Though their vision may not focus on the same spot during the trial, any trial with obvious head movements (e.g. looking around) was excluded from analysis.

In conclusion, some age-related changes in postural control were observed in children with ASD. The U14 group exhibited improved mediolateral postural stability compared to U8, whereas no differences were found between U8 and U11 or between U11 and U14. These findings may suggest that children with ASD may slowly develop postural stability but only demonstrate significant changes over a long period of time. We recommend early intervention programs specifically focused on improving complexity of postural control as potentially beneficial for children with ASD. Future research is warranted to investigate postural control complexity for young autistic children and evaluate the efficacy of early interventions to enhance postural stability.
